# Protocol for generating protein profiles and distance-based network analysis of murine tissue slices

**DOI:** 10.1016/j.xpro.2024.103578

**Published:** 2025-01-20

**Authors:** Luisa Schmidt, Philipp Antczak, Marcus Krüger

**Affiliations:** 1 Institute for Genetics, Cologne Excellence Cluster on Cellular Stress Responses in Aging-Associated Diseases (CECAD), 50931 Cologne, Germany; 2 Center for Molecular Medicine Cologne (CMMC), University of Cologne, 50931 Cologne, Germany; 3 Department II of Internal Medicine, University of Cologne, Faculty of Medicine and University Hospital Cologne, Cologne, Germany

**Keywords:** Bioinformatics, Cell Biology, Protein Biochemistry

## Abstract

We introduce a protocol for spatial proteomics using thin cryotome sections of mouse skeletal muscle tissue. We describe steps for preparing muscle sections and liquid chromatography-tandem mass spectrometry (LC-MS/MS) analyses to generate spatial protein profiles along the longitudinal skeletal muscle axis. We detail procedures for scanning longitudinal protein profiles and replacing missing data using a sliding window approach. This protocol has potential for applications in spatial proteomics to other tissues with asymmetric patterns such as the brain and heart tissue.

For complete details on the use and execution of this protocol, please refer to Schmidt et al.^1^

## Highlights

• Protocol for generating protein profiles based on LC-MS/MS data from tissue sections
• Simple steps for sample preparation for LC-MS/MS analysis from tissue sections
• Detailed instructions for data analysis to generate protein profiles
• Precise guidelines for network generation of protein profiles

## Abstract (editor highlights)

Publisher’s note: Undertaking any experimental protocol requires adherence to local institutional guidelines for laboratory safety and ethics.

## Graphical abstract

**Figure:**
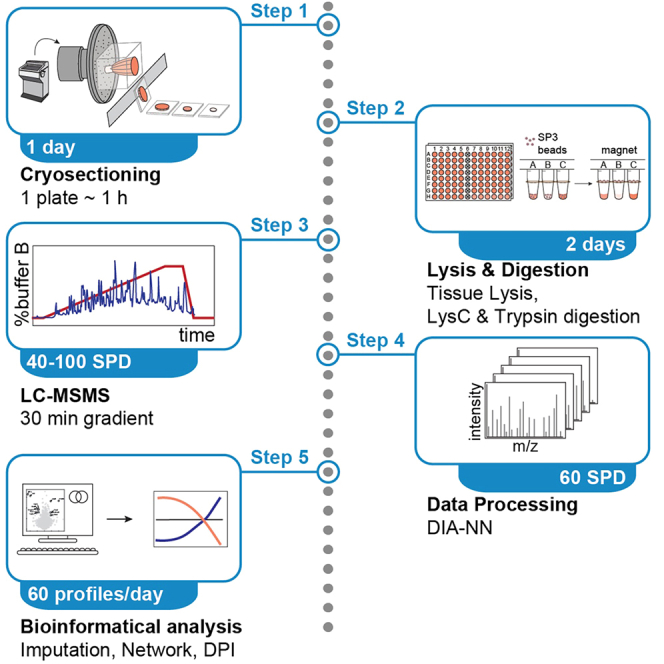

## Before you begin

We established a method for spatial mapping of proteins in skeletal muscle fibers, the myotendinous junction (MTJ), and tendons that are not biased by morphological features. This method is particularly well-suited for spatially characterizing tissue along the longitudinal axis over several millimeters. This is particularly important for the analysis of junctions such as the MTJ or tissues with different layers, where each layer has its own structure, cellular composition and function. A similar method that has recently been developed is micro-scaffold assisted spatial proteomics (MASP), which also provides detailed insights into the distribution and organization of proteins in biological structures. In MASP the spatial resolution of the tissue is achieved using brain tissue sections, a 3D-printed piston array, followed by mass spectrometric analysis.^2^ The following protocol describes the steps required to use cryo-embedded mouse soleus muscles for spatial proteomics profiling. We describe the process of the soleus muscle isolation from the mouse hind leg and analyze single thin cryosections with a thickness of 20 μm using a cryotome. The section thickness of the samples can be adjusted based on the desired resolution of the protein profiles. In addition, parameters such as the sample’s surface area, the type of embedding used, and the sensitivity of the mass spectrometer play a crucial role.

Each section is placed in a v-shaped 96-well-plate and the extracted proteins are subjected to tryptic protein digestion using the single-pot, solid-phase-enhanced sample-preparation (SP3) protocol. Each section is analyzed using short LC-MS gradients to generate spatial protein profiles along the longitudinal axis between muscle fibers and tendon. The bioinformatics approach involves an alternative normalization for samples with input variances which cannot be covered by normal label-free quantitation (LFQ) normalizations, and a sliding window scans an area of 200 μm to filter data and impute missing values. Missing values are generated due to tissue heterogeneity between different tissue layers and areas, sectioning issues, and LC-MS/MS limitations in protein dynamic ranges and abundances. The profiles are then subjected to distance-delay analysis to create a distance-based network. Distance-delay analysis describes the dependence between the proteins localization within the tissue to understand the biological relevance and protein connections.

In summary, this workflow can be used to generate protein profiles for various muscle structures and other tissues, and thus contributes to development of spatial repositories of organs and improve our understanding of structural changes and protein localizations in disease models.

### Muscle extraction

**Timing: 10 min per tissue**

This section describes the preparation of the isolated tissue. Here, we used the soleus skeletal muscle of the mouse hindlimb.

**Figure 1:**
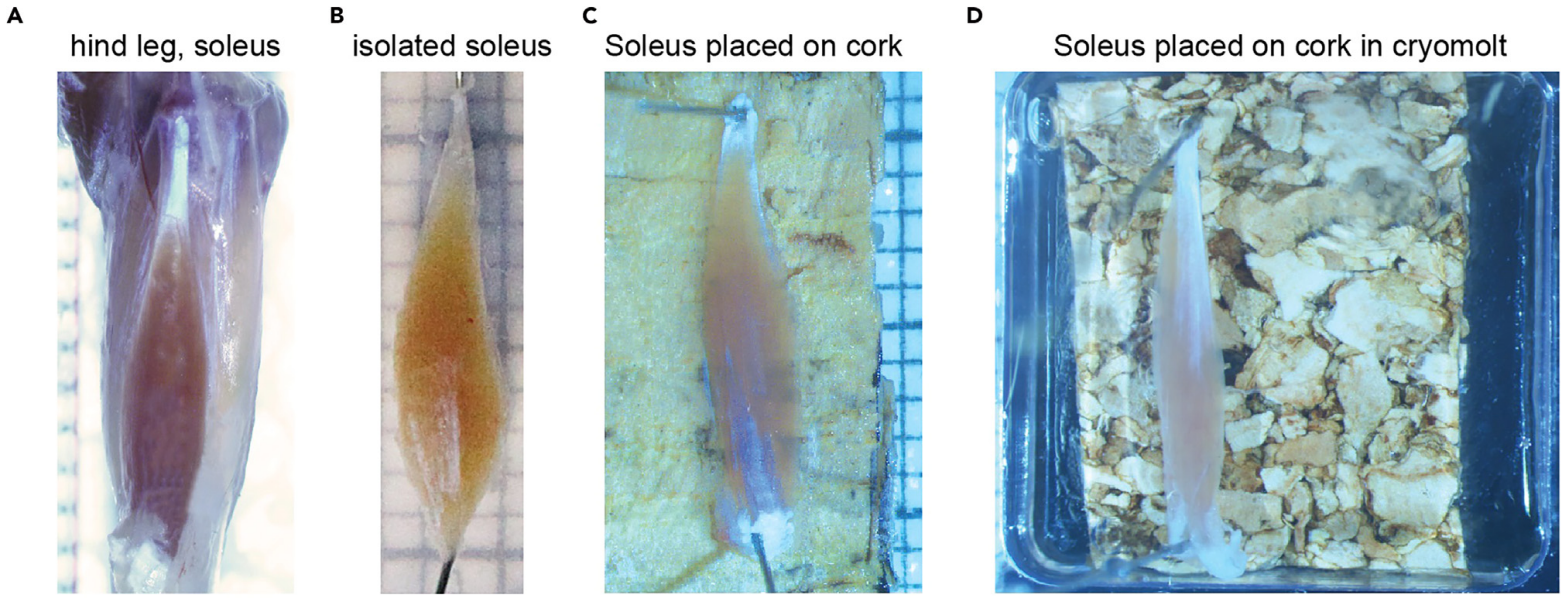
Isolation and attachment of the mouse soleus muscle (A) Intact mouse soleus skeletal muscle of the hind leg of the mouse. (B) Isolated soleus muscle is placed on transparent silicone for morphometric visualization. (C) The mounted muscle is transferred to a cork base and fixed in position using insect needles. (D) The mounted muscle and cork are transferred to a cryomolt, which is filled with cryomatrix and frozen.
3. Attach the skeletal muscle to a transparent silicone mat with insect needles to document the morphology.
4. Size of the muscle using a stereomicroscope and camera (Figure 1B).
5. Transfer the cork mat and the skeletal muscle to a cryomold.
6. Fill the cryomold with cryomatrix (Tissue-Tek O.C.T. compound) until the muscle is completely covered by the matrix (Figure 1D).
7. Freeze the cryomolds on dry ice for 10 min.
8. Remove the needles from the fixated tissue.
9. Store the samples at −80°C.

***Note:*** To prevent muscle contraction, we attached the soleus to the cork mat with small needles (Figure 1C).

1. House adult male wild-type C57BL/6 mice at 22° ± 2°, 55 ± 10% humidity, and an air exchange rate of 15 times per hour under a 12:12 h light-dark cycle with free access to standard chow diet.
2. Isolate a skeletal muscle of interest from the hind leg of the mouse or another area of the body (Figure 1A).

## Key resources table

**Table:** 

REAGENT or RESOURCE	SOURCE	IDENTIFIER
**Chemicals, peptides, and recombinant proteins**		

Lysyl endopeptidase	Wako	Cat# 129-02541
Sequencing grade modified trypsin	Serva	Cat# 37286.03
Internal retention time (iRT) peptides	GeneCust	NA
Ammonium bicarbonate (ABC)	Merck	207861
Tris(2-carboxyethyl)-phosphine, hydrochloride (TCEP)	Thermo Fisher Scientific	77720
Ammonium hydroxide solution (NH_4_OH)	VWR	470300-212
2-Chloroacetamide (CAA)	Sigma	C0267-100G
Formic acid (LC grade)	VWR	64-18-6
Acetonitrile (LC grade)	VWR	83640.320
Acetonitrile + 0.1% FA (LC grade)	VWR	84866.290
Water (LC grade)	VWR	83645.320
Water + 0.1% formic acid (FA) (LC grade)	VWR	84867.320
Methanol (LC grade)	VWR	20847.320
Ethanol (>99.5%)	VWR	85033.360
SDS	VWR	151-21-3
Sera Mag beads A	VWR	44152105050250.
Sera Mag beads B	VWR	65152105050250
Tissue-Tek	Laborversand.de	TTEK

**Deposited data**		

C57BL/6 untargeted proteomics	Schmidt et al.^1^	ProteomeXchange (http://www.proteomexchange.org) https://www.ebi.ac.uk/pride/archive/projects/PXD046596
Code of the spatial analysis (sliding window for filter, imputation, k-means clustering, distance-delay analysis)	Schmidt et al.^1^	Zenodo (https://zenodo.org/) https://doi.org/10.5281/zenodo.10678005

**Experimental models: Organisms/strains**		

Mice: C57BL/6, male, 2-month, wild type	The Jackson Laboratory	Cat# 000664

**Software and algorithms**		

R (v.4.3.3)	The R Project	https://www.r-project.org; RRID: SCR_001905
Rstudio	RStudio	https://www.rstudio.com; RRID:SCR_000432
ProteoWizard (v.3.0.21218)	ProteoWizard	http://proteowizard.sourceforge.net; RRID:SCR_012056
Freestyle software suite	Thermo Fisher Scientific	RRID:SCR_022877
Skyline Daily (v.22.2.1.391)	Skyline	https://skyline.ms/project/home/software/skyline/begin.view RRID:SCR_014080
DIA-NN	Demichev, 2019 https://www.nature.com/articles/s41592-019-0638-x	https://github.com/vdemichev/DiaNN; RRID:SCR_022865
Cytoscape	Cytoscape Consortium	http://cytoscape.org; RRID:SCR_003032
R package: samr	–	NA
R package: vsn	–	RRID:SCR_001459
R package: ggplot2	–	RRID:SCR_014601
R package: gplots	–	RRID:SCR_025035
R package: slider	–	NA
R package: openxlsx	–	RRID:SCR_019185
R package: preprocessCore	–	RRID:SCR_024254
R package: pbapply	–	NA
R package: igraph	–	RRID:SCR_019225
R package: parallel	–	NA
R package: devtools	–	RRID:SCR_016961
R package: diann	–	NA

**Other**		

Orbitrap Eclipse Tribid	Thermo Fisher Scientific	RRID:SCR_023618
Faims Pro	Thermo Fisher Scientific	NA
Ultimate 3000 HPLC system	Thermo Fisher Scientific	NA
Cryotome	Reichert Jung/Leica	NA
Tissue-Tek Cryomold	Sakura	(15 mm × 15 mm x 5 mm)
Cork	Kork-Deko.de	NA
Needles	Bioform	Gr. 000 (0.25 × 38 mm)
v-shaped 96-well plates	Thermo Fisher Scientific	AB-2396
Silicone sealing mat	Nerbe Plus	04-090-0000
Zone free sealing films	Merck	Z721646-50EA
One-sided plate magnet	NIPPON Genetics EUROPE	FG-SSMAG96
Styrene divinylbenzene-reverse phase solid (SDB-RPS)	Merck	66886-U
Bioruptor Pico sonication device	Diagenode	B01060010
SpeedVac	Eppendorf	Concentrator Plus
Fused silica emitter (20 μm)	MS Wil	1893528
8 cm PepSep column	MS Wil	1893470

## Materials and equipment

**Table:** PBS with MilliQ water (phosphate buffered saline, 1X, pH 7.4)

Reagent	Final concentration	Amount
Sodium chloride (mw: 58.44 g/mol)	0.137 M	8 g
Potassium chloride (mw: 74.55 g/mol)	0.0027 M	0.2 g
Sodium phosphate dibasic (mw: 141.96 g/mol)	0.01 M	1.44 g
Potassium phosphate monobasic (mw: 136.09 g/mol)	0.0018 M	0.245 g
**Total**	**N/A**	**1 L**

The buffer can be stored for one month at RT.

### Stock solutions

4% SDS in PBS.

 Buffer can be stored for one month at RT.

ABC buffer: 50 mM ABC in MilliQ water (pH 8.0).

 Buffer can be stored for one month at 4°C.

550 mM 2-Chloroacetamide (CAA) in MilliQ water.

0.5 μg/μL Trypsin protease (dilute in MilliQ water).

0.5 μg/μL LysC protease (dilute in MilliQ water).

 All of these stock solutions can be aliquoted and stored for a year at −20°C.

**CRITICAL:** SDS, CAA, and TCEP are toxic. Pipette these chemicals in a fume hood and handle them with gloves.

Bead solution: Mix 20 μL of Sera Mag beads A and 20 μL of Sera Mag beads B. Wash the beads three times with 200 μL water (LC grade), using a magnet to immobilize the beads in the tube after each wash step. Reconstitute the beads in 200 μL of water.

Lysis buffer: 5 mM TCEP and 15 mM CAA in the 4% SDS in PBS buffer.

**CRITICAL:** The lysis buffer must be freshly prepared before the experiments.

Digestion buffer: 20 ng of trypsin and 10 ng of LysC in ABC buffer.

**CRITICAL:** buffer must be prepared freshly.

Buffer A: 0.1% Formic acid in MilliQ water.

Buffer B: 0.1% Formic acid in 80% acetonitrile in MilliQ water.

 Buffers can be stored for one month at RT.

Buffer R: iRT peptides and 2% formic acid in 5% acetonitrile in MilliQ water.

 Buffer can be stored for one month at 4°C.

Elution Buffer: 1% ammonia in 60% acetonitrile in MilliQ water.

 Buffer can be stored for two weeks at RT.

**CRITICAL:** Formic acid vapor can severely irritate the eyes, mucous membranes, and skin. When preparing this buffer, pipette under fume hood and wear gloves.

## Step-by-step method details

### Cryotome sectioning

**Timing: ∼1 h per plate**

This section describes the sectioning of the tissue of interest using a cryotome followed by transferring each slice to a 96-well plate.

1. Attach the embedded sample to the rotary microtome of the cryotome using cryomatrix. Align the block at a right angle to the blade (Figure 2A) (problem 1).

**Figure 2:**
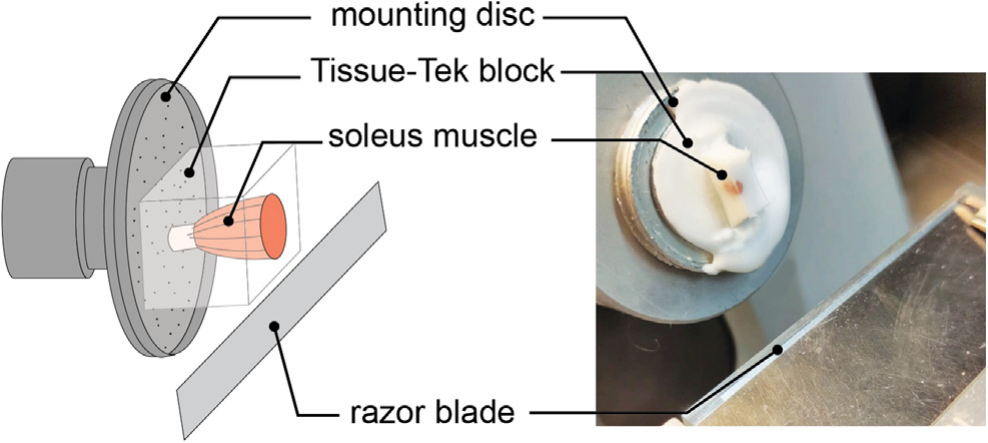
Correct positioning of the tissue on the mounting disk for cryosectioning
2. Cut the embedded tissue sample:
  a. Trim the sample block with a razor blade in order to transfer as little of the surrounding cryomatrix as possible when the sections are cut.
  b. Cut the block until the embedded tissue becomes visible - this will be the first section to be used for analysis (Figure 3A).

**Figure 3:**
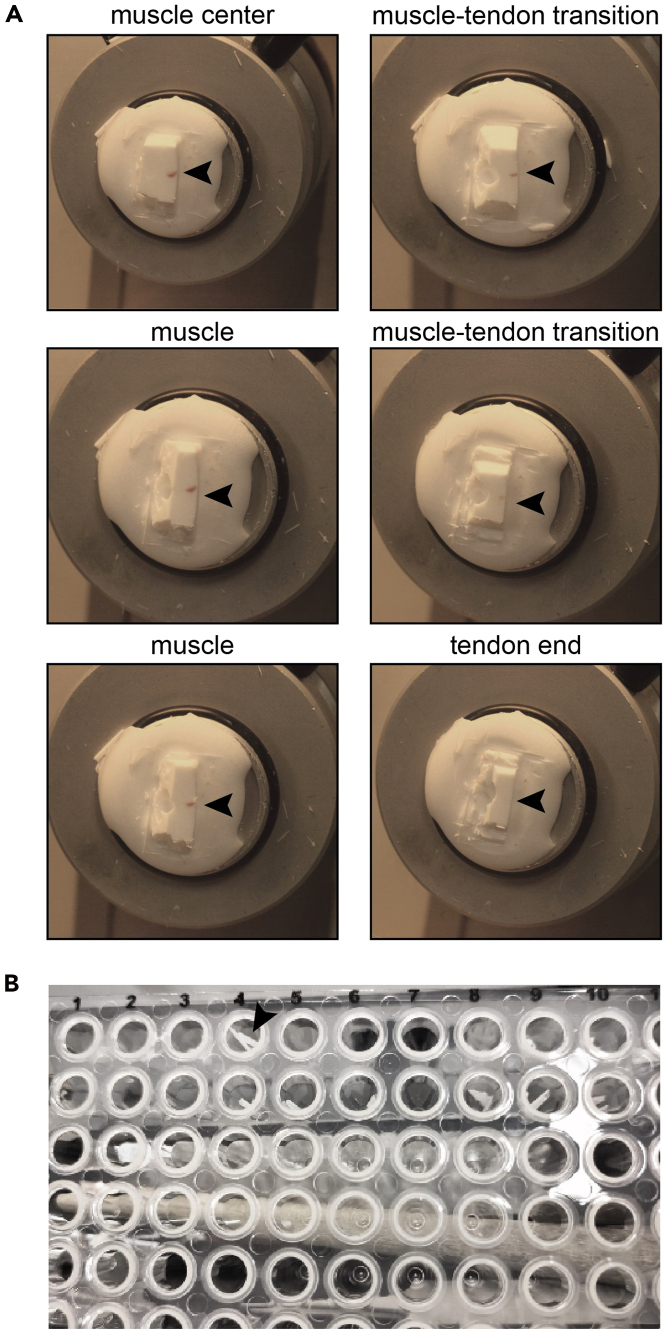
Tissue sampling (A) Tissue-Tek embedded muscle, after cryosectioning from the central muscle to the tendon end. (B) Image of 20 μm-thick sections placed in a 96-well-plate.
  c. Cut the embedded tissue from the central to the peripheral regions at a thickness of 20 μm (Figure 3A).
  d. Transfer each slice into a v-shaped 96-well-plate using cooled tweezers (Figure 3B) (problem 2).
  e. Seal the 96-well plate with a silicone sealing mat.

***Note:*** One column of the 96-well plate should not be filled with sections as it will be used for control samples.

***Note:*** Tissue sections can be cut into thinner or thicker sections to increase or decrease the resolution of the analysis. The choice of slice thickness depends on the area/size of the tissue and the sensitivity of the MS instrument and should be individually determined for each tissue.

**CRITICAL:** All materials for cutting 96-well plates, tweezers, and cryo-embedded samples should be chilled at −20°C for at least 30 min before use. Insufficient cooling of the materials can lead to thawing of the tissue and sample loss. Importantly, the 96-well plates should also be kept in the cryotome at −20°C during sample collection.

**Pause point:** Samples can be stored at −20°C until further use.

### Tissue lysis and digestion

**Timing: 2 days**

In this section the tissue slices are lysed, reduced, and alkylated. Protein lysates are tryptic digested using the single-pot, solid-phase-enhanced sample-preparation (SP3) approach and prepare for LC-MS/MS analysis.

3. Tissue lysis.
  a. Add 40 μL lysis buffer to each well.
  b. Fill empty control wells with 40 μL of human embryonic kidney 293 (HEK-293) cell lysates in the same lysis buffer.
  c. Heat the 96-well-plates to 95°C for 5 min and centrifuge at 248 g for 5 min.
  d. Sonicate the 96-well plates for 10 min in a Bioruptor system using an on/off interval of 30 s.
  e. Heat the 96-well-plates to 70°C for 10 min.
  f. Centrifuge for 5 min at 248 g.

***Note:*** Instead of HEK cells, any other cell line can be used as quality control for digestion and sample preparation.

**Pause point:** Samples can be stored at −20°C until further use. If the samples are stored, repeat 3c, and 3e.

4. SP3 precipitation.
  a. Add 2 μL of the Sera-Mag bead mixture to each sample.
  b. Add 42 μL of acetonitrile and vortex carefully.
  c. Incubate the 96-well-plates for 8 min at room temperature.
  d. Place the 96-well plate on a one-sided plate magnet for 2 min.
  e. Remove the supernatant carefully.
  f. Wash the 96-well-plates twice with 100 μL of 70% ethanol
  g. Wash the 96-well-plates once with 100 μL of 100% acetonitrile.
  h. Remove the 96-well-plates from the magnet and air dry for 2 min.
5. Protein digestion with LysC and trypsin.
  a. Add 10 μL digestion buffer to each well.
  b. Incubate samples overnight at 27°C (problem 3).
  c. Add 100 μL buffer A to stop protein digestion.

**CRITICAL:** The beads must be completely reconstituted in digestion buffer. DO NOT pipette up and down, as the beads will adhere to the pipette.

6. Sample purification and desalting with StageTips.
  a. Place two layers of styrenedivinylbenzene reverse-phase sulfonate (SDB-RPS) on top of each other and punch them out with the help of a modified syringe as described in Rappsilber et al.^3^^,^^4^
  b. Press the two double layers out of the syringe into a 200 μL pipette tip.
  c. Add 50 μL methanol to equilibrate the SDB-RPS stop and go extraction tips (StageTips) and centrifuge at 800 g for 1 min.^3^
  d. Wash the StageTips with 50 μL buffer B and centrifuge at 800 g for 1 min.
  e. Wash the StageTips twice with 50 μL buffer A and centrifuge at 800 g for 1 min.
  f. Pipette the samples on top of the StageTips and centrifuge at 400 g for 5 min.
  g. Wash the StageTips with 100 μL buffer A and centrifuge at 800 g for 1 min.
  h. Wash the Stage Tips twice with 50 μL buffer B and centrifuge at 800 g for 1 min.
  i. Dry the StageTips by centrifugation at 800 g for 10 min.

**Pause point:** Samples can be stored at 4°C until further use.

7. Sample elution.
  a. Place the StageTips in a tips-to-well adapter placed on a 96-well plate suitable for LC-MS/MS.
  b. Add 30 μL elution buffer to each StageTip.
  c. Place the plate with the StageTips in a centrifuge equipped with an adapter for 96-well plates that are suitable for your LC-MS system.
  d. Quickly spin down the elution buffer in the StageTips for 10 s to incubate the loaded SDB-RPS material with the elution buffer.
  e. Incubate the StageTips with the elution buffer for 30 min.
  f. Centrifuge the StageTips at 400 g for 5 min.
  g. Place the 96-well plates in the SpeedVac at RT for 45 min to remove the elution buffer.
  h. Reconstitute the peptides with 6 μL buffer R.
  i. Seal the 96-well plate with a zone free sealing film.

**Pause point:** Samples can be stored at −20°C until further use.

### LC-MS/MS analysis

**Timing: 40–100 samples per day (SPD)**

This section describes the LC-MS/MS setup for proteome analysis using an Ultra-High-Performance Liquid Chromatography system (UHPLC Ultimate 3000) coupled to a quadrupole-Orbitrap mass spectrometer (Eclipse Tribid).

8. The setup and usage of LC-MS/MS instrumentation should be conducted according to the manufacturer’s instructions.
  a. Add indexed retention time peptides (iRT) to each sample and monitor the iRT
       Peptides via Skyline Daily (V 22.2.1.391) to ensure stable LC performance.
  b. Inject 4 μL of each reconstituted peptide sample into an 8 cm PepSep column (150 μm inner diameter, 1.5 μm particle size). Analyze the sample using an Ultimate 3000 (Thermo-Fisher Scientific, Waltham, USA) with an integrated column oven coupled to an Orbitrap Eclipse Tribid mass spectrometer equipped with an FAIMS-Pro interface (Thermo-Fisher Scientific, Waltham, USA).
  c. Inject a blank sample (buffer A) every 15th sample as a control for carry over.

**Table:** 

iRT peptide	*m/z (z* = 2)	Mass [mol]	iRT peptide	*m/z* (*z* = 2)	Mass [mol]
LGGNEQVTR	487.2567	2.26E-06	TPVISGGPYEYR	669.8381	1.57E-06
GAGSSEPVTGLDAK	644.8226	1.78E-06	DGLDAASYYAPVR	699.3384	1.57E-06
TPVITGAPYEYR	683.8537	1.83E-06	YILAGVENSK	547.298	2.38E-06
VEATFGVDESNAK	683.8279	1.83E-06	GTFIIDPGGVIR	622.8535	1.77E-06
LFLQFGAQGSPFLK	776.9298	1.42E-06	ADVTPADFSEWSK	726.8357	1.51E-06
GTFIIDPAAVIR	636.8692	1.89E-06	–	–	–
  d. Elute the peptides from the column using mobile phases A (0.1% formic acid in LC-grade water) and B (0.1% formic acid in 80% LC-grade acetonitrile) keeping the column at a constant temperature of 50°C.
  e. Set the LC parameters as follows:

**Table:** 

Elution mode	One column separation
Separation column	PepSep fused silica emitter (20 μm) connected to an 8 cm PepSep column (150 μm, 1.9 particle size)
Column temperature	50°C
Faims CV	−50 V
    i. LC gradient parameters.

**Table:** 

Time [min]	Duration [min]	Composition (%B)	Flow rate (μL/min)
0	0	3	450
1:30	1:30	3	450
2:30	1:00	8	450
23:30	21:00	30	400
26:00	2:30	55	400
26:30	0:30	95	400
31:00	4:30	95	400
32:00	1:30	3	400
34:30	2:30	3	–
  f. Set the MS parameters as follows:
    i. MS Global Settings.

**Table:** 

Application mode	Peptide
Method Duration [min]	35
Infusion Mode	Liquid chromatography
Expected LC peak width (s)	8
Default charge state	2
    ii. FAIMS Settings.

**Table:** 

FAIMS inner electrode temp [°C]	99.5
FAIMS outer electrode temp [°C]	85.5
Total carrier gas flow	Static
Total carrier gas flow [L/min]	3.7
    iii. MS1 scan.

**Table:** 

Detector type	Orbitrap
Orbitrap resolution	15,000
Scan range [*m/z*]	380–900
RF lens [%]	40
Normalized AGC target %	250
Maximum injection time [ms]	20
Data type	Centroid
Polarity	Positive
    iv. First DIA scan.

**Table:** 

Precursor Mass range [*m/z*]	400–850
Isolation mode	Quadrupole
Isolation window [*m/z*]	15
Window overlap [*m/z*]	2
Number of scan events	30
Activation type	HCD
HCD collision energy	Normalized
HCD collision energy [%]	31
Detector type	Orbitrap
Orbitrap resolution	15,000
Scan range [*m/z*]	250–1700
RF Lens [%]	40
Normalized AGC target %	1000
Maximum injection time [ms]	25
Data Type	Centroid
Polarity	Positive
    v. DIA m/z windows.

**Table:** 

*m/z* range	*m/z* range	*m/z* range	*m/z* range
399.4314–416.4392	519.486–536.4937	639.5406–656.5483	759.5952–776.6029
414.4383-431-446	534.4928–551.5006	654.5474–671.5551	774.602–791.6097
429.4451–446.4528	549.4997–566.5074	669.5542–686.562	789.6088–806.6165
444.4519–461.4596	564.5065–581.5142	684.561–701.5688	804.6156–821.6233
459.4587–476.4665	579.5133–596.521	699.5679–686.562	819.6224–836.6302
474.4656–491.4733	594.5201–611.5297	714.5747–731.5824	834.6293–851.637
489.4724-506-4801	609.5269–626.5347	729.5815–746.5829	–
504.4792–521.4869	624.5338–641.5415	744.5883–761.5961	–
    vi. MS1 scan as described before.
    vii. Second DIA scan.

**Table:** 

Precursor mass range [*m/z*]	392.5–857.5
Isolation mode	Quadrupole
Isolation window [*m/z*]	15
Window overlap [*m/z*]	2
Number of scan events	30
Activation type	HCD
HCD collision energy	Normalized
HCD collision energy [%]	31
Detector type	Orbitrap
Orbitrap resolution	15,000
Scan range [*m/z*]	250–1700
RF lens [%]	40
Normalized AGC target %	1000
Maximum injection time [ms]	25
Data type	Centroid
Polarity	Positive
    viii. DIA m/z windows.

**Table:** 

*m/z* range	*m/z* range	*m/z* range	*m/z* range
391.4278–408.4355	511.4824–528.4901	631.5369–648.5447	751.5915–768.5992
406.4346–423.4424	526.4892–543.4696	646.5438–663.5515	766.5983–783.6061
421.4414–438.4492	541.496–558.5038	661.5506–678.5583	781.6052–798.6129
436.4483–453.456	556.5028-573-5106	676.5574–693.5651	796.612–813.6197
451.4551–468.4628	571.5097–588.5174	691.5642–708.572	811.6188–828.6265
466.4619–483.4696	586.5165–603.5242	706.5711–723.5788	826.6256–843.6334
481.4687–498.4765	601.5233–618.531	721.5779–738.5856	–
496.4756–513.4833	616.5301–633.5379	736.5847–753.5924	–

### Data processing

**Timing: 60 samples per day**

Acquired data-independent acquisition (DIA) spectra were processed with analysis software to calculate peptide abundances.

9. Process the acquired DIA spectra using software packages such as DIA-NN,^5^ MaxQuant,^6^ or Spectronaut^7^ according to the instructions.
  a. Convert RAW data into ‘.dia’ format.
  b. Set the following parameters for RAW data analysis: digestion with trypsin/P and one missed cleavage. Select a current UniProt FASTA database (we used UniProt *Mus musculus* database Sep. 2017).
  c. Adjust precursor and fragment ion *m/z* range according to the MS settings; typical *m/z* ranges would be 400–1000 and 350–1600, respectively.
  d. Create the main output of the data and the output library in the correct directory.
  e. Set the precursor FDR to 1% and select the thread settings according to the available cores.
  f. Mass accuracy is calculated by DIA-NN.
  g. Set ‘MBR’, ‘Heuristic protein inference’, and ‘No shared spectra’ as active.
  h. Choose ‘protein names (from FASTA)’ for protein inference settings.
  i. Choose ‘double-pass mode’ for the neural network classifier.
  j. Choose ‘high precision’ for the quantification strategy.
  k. Set Cross-run normalization, Library generation, and Speed and RAM to the default parameters.
  l. For additional information add ‘--report-lib-info’ option.

***Note:*** The time required to process the data highly depends on the number of samples, LC-MS instrument, gradient length, and the CPU.

### Bioinformatic analysis: Generation of protein profiles

**Timing: 120 profiles per day**

This chapter describes the generation of protein profiles of the acquired LC-MS/MS data from sequential tissue sections. Original data and code are provided on https://www.ebi.ac.uk/pride/archive/projects/PXD046596 and https://doi.org/10.5281/zenodo.10678005.

10. Data preprocessing.
  a. Process the data output from DIA-NN with the recommended R script from https://github.com/vdemichev/diann-rpackage,^5^ which is called here ‘Dataset1’.
  b. Order the Dataset1 after the experiment number and sections, which is called here ‘Dataset1_sorted’.
  c. Read and subset the data to normalizing strategies. Dataset1_sorted.xlsx is provided together with the original code on Zenodo: https://doi.org/10.5281/zenodo.10678005.
    > install.packages(“samr”)
    > install.packages(“vsn”)
    > install.packages(“ggplot2”)
    > install.packages(“gplots”)
    > install.packages(“slider”)
    > install.packages(“openxlsx”)
    > install.packages(“preprocessCore”)
    > library(samr)
    > library(vsn)
    > library(ggplot2)
    > library(gplots)
    > library(slider)
    > library(openxlsx)
    > library(preprocessCore)
    > ### Data reading and preprocessing
    > #read data and subset columns
    > data <- read.xlsx("Dataset1_sorted.xlsx")
    > data.cols <- sapply(strsplit(colnames(data),"_"),function(x) x[1])
    > #generate data subsets
    > data.x <- list(
     GENES=data[,which(data.cols == "GENES")],
     GENESnorm=data[,which(data.cols == "GENESnorm")],
     GENESquant=data[,which(data.cols == "GENESquant")],
     MaxLFQ=data[,which(data.cols == "MaxLFQ")],
     PG.Norm=data[,which(data.cols == "PG.Norm")],
     PG.Quant=data[,which(data.cols == "PG.Quant")],
     rest=data[,grep("ˆS",data.cols)])
  d. Read the metadata out of the data column names. The biological replicate of the sectioned tissue is called “replicate” (original code: soleus), and the consecutive sections is called “slice”.
  e. Normalize data to the sum intensity in each slice (problem 4).
    > #extract the PG Quant data, preprocess, and normalize data
    > tmp <- as.matrix(data.x$PG.Quant)
    > #ensure that table is a matrix
    > tmp[which(!is.finite(tmp))] <- NA
    > #remove any value that is not finite i.e. Inf or NaN
    > tmp[which(tmp < 1e4)] <- NA
    > #set low intensity measures to NA
    > rownames(tmp) <- data$Protein.Group
    > cannot <- data.frame(id=colnames(tmp),replicate=sapply(strsplit(colnames(tmp),"_"),function(x) x[2]),slice=sapply(strsplit(colnames(tmp),"slice"),function(x) as.numeric(x[length(x)])))
    > rownames(cannot) <- cannot$id
    > cannot$int <- colSums(tmp,na.rm=T)
    > #calculate total remaining intensity
    > tmp <- tmp[,which(cannot$int != 0)]
    > #ensure all retained samples have an intensity
    > cannot <- cannot[which(cannot$int != 0),]
    > #subset column annotation to same size of dataset
    > tmp.quant <- justvsn(as.matrix(tmp))
    > #VSN normalize the data
    > tmp.norm <- tmp.quant-log2(cannot$int)
    > #correct for total intensity
    > ### Data reading and preprocessing END
11. Introduce a sliding window to evaluate missing values (problem 5).

> ### Evaluating missings

> data.full.s <- tmp.norm

> cannot.s <- cannot

> #calculate sample specific missing values

> data.col.na <- apply(data.full.s,2,function(x) length(which(is.na(x)))/length(x))

> #generate sliding window NA count

> slideNA <- tapply(rownames(cannot.s),cannot.s$replicate,function(x){

naprot <- apply(data.full.s[,x],1,function(z){

return(slide_dbl(z,function(k) length(which(is.na(k)))/length(k),.after=10,.complete=T))})

return(naprot)})

> #select proteins based on sliding Window and a 70% data rate

> selectedProteins <- names(which(rowMeans(sapply(slideNA,function(x) apply(x,2,min,na.rm=T))) <= 0.3))

> ### Evaluating missings END

12. Impute missing values (problem 6).
  a. Impute all missing values using the sliding window.
    > ### Imputing missing values
    > #imputing values across a spatial profile using sliding windows
    > imputeProteomics_byDistance <- function(datax,annot,slice.width.forward=10,slice.width.backward=10,newfit.na="impute",sel=0.05, m.offset=1.6,sd.offset=0.8, min.data.required=3){
    annot <- annot[colnames(datax),]
    newdatx <- by(t(datax),annot$replicate,function(x){
    .pos <<- x
    slice <- annot[rownames(x),"slice"]
    slicex <- seq(min(slice),max(slice),by=1)
    names(slicex)[match(slice,slicex)] <- rownames(x)
    newx <- apply(x,2,function(z){
    .pos2 <<- z
    names(z) <- rownames(x)
    if(any(is.na(z))){allx <- slide(slicex,∼list(start=.x[1],stop=.x[length(.x)],pos=length(na.omit(names(.x)))),.before=slice.width.backward,.after=slice.width.forward,.complete=F)
    allx <- allx[which(!is.na(names(allx)))]
    allxs <- data.frame(start=sapply(allx,function(x) x$start),stop=sapply(allx,function(x) x$stop),len=sapply(allx,function(x) x$pos))
    rownames(allxs) <- names(allx)
    allx <- subset(allxs,len > min.data.required)
    allx <- allx[names(which(is.na(z))),]
    allx <- subset(allx,!is.na(start))
    allx$pos_real <- rownames(allx)
    if(nrow(allx) > 0){
    newfits <- apply(allx,1,function(l){
    .pos3 <<- l
    pos <- na.omit(names(slicex[match(as.numeric(l[1]):as.numeric(l[2]),slicex)]))
    tmpf <- data.frame(prot=z[pos],annot[pos,])
    tmp <- subset(tmpf,!is.na(prot))
    if(nrow(tmp) >= min.data.required){
    lmr <- lm(prot∼slice,data=tmp)
    pred <- predict(lmr,newdata=data.frame(slice=tmpf$slice))
    pred[pred < min(x,na.rm=T)] <- NA
    names(pred) <- rownames(tmpf)
    return(pred[l["pos_real"]])}else{
    if(newfit.na == "impute"){
    zr <- range(z,na.rm=T)
    xx <- as.matrix(x)
    xm <- xx[which(xx > zr[1] & xx < zr[2])]
    return(rnorm(1,mean=mean(xm,na.rm=T)-m.offset∗sd(xm,na.rm=T),sd=sd(xm[which(xm < quantile(xm,probs=sel,na.rm=T))],na.rm=T)∗sd.offset))
    }else{return(NA)}}})
    names(newfits) <- rownames(allx)
    z[names(newfits)] <- newfits
    return(z)}else{return(z)}}else{return(z)}})
    return(newx)})
    newdatx <- do.call(rbind,newdatx)
    return(t(newdatx))}
  b. Use only proteins with enough data points.
    > #only use proteins with enough data
    > data.norm <- data.full.s[match(selectedProteins,rownames(data.full.s)),]
    > #impute by distance and a simple linear model, forward and backward slices tell it "how far to go" on a "per slice basis" (not on a per available sample basis)
    > data.norm <- imputeProteomics_byDistance(data.norm,cannot.s,slice.width.forward=10, slice.width.backward=10,min.data.required=3,newfit.na="none")
    > #impute whatever couldn't be imputed to the lower 5% of the data
    > data.norm <- perseus.impute(data.norm)
    > data.norm <- data.norm[,rownames(cannot.s)]
    > ### Imputing missing values END
13. Bootstrap analysis to compress or stretch biological replicates.
  a. Identification of proteins that change in abundance over the tissue (problem 7).
    > ### Evaluate the need for compression ratios between samples
    > #identify proteins that change across slice
    > res <- lapply(unique(cannot.s$replicate),function(x){
    tmp <- subset(cannot.s, replicate == x)
    dat.tmp <- data.norm[,rownames(tmp)]
    data.sam <- list(x=dat.tmp,y=tmp$slice,genenames=rownames(dat.tmp),geneid=rownames(dat.tmp),logged2=T)
    samr.obj <- samr(data.sam,resp.type="Quantitative",nperms=1000)
    delta.table <- samr.compute.delta.table(samr.obj)
    sig <- samr.compute.siggenes.table(samr.obj,1,data.sam,delta.table,all.genes=T)
    return(list(data=data.sam,samr=samr.obj,delta.table=delta.table,sig=sig))})
    names(res) <- unique(cannot.s$replicate)
    > scores <- lapply(lapply(res,function(x) rbind(x$sig$genes.up,x$sig$genes.lo)),function(x) { tmp <- as.numeric(x[,4]); names(tmp) <- x[,2]; return(tmp)})
    scores <- do.call(cbind,lapply(scores,function(x) x[order(names(x))]))
    > #assign new variables to evaluate different thresholds
    > data.final <- data.norm[,rownames(cannot.s)]
    > scores2 <- scores
    > #identify which proteins follow similar trajectories between samples
    > scores2.s <- scores2[,as.character(unique(cannot.s$replicate)),drop=F]
    scores2.s <- cbind(scores2.s,abs(apply(scores2.s,1,function(x) sum(diff(x)))))
    > scores2.sm <- data.frame(mean=apply(scores2.s[,-ncol(scores2.s)],1,mean),absdiff=abs(apply(scores2.s[,-ncol(scores2.s)],1,function(x) sum(diff(x)))))
    > scores2.sm$fscore <- scores2.sm[,1]∗(1/scores2.sm[,2])
    > scores2.smo <- scores2.sm[order(abs(scores2.sm$fscore),decreasing=T),]
    > cannot.s2 <- do.call(rbind,by(cannot.s,cannot.s$replicate,function(x){ x$perc <- (x$slice2-min(x$slice2))/(max(x$slice2)-min(x$slice2)); return(x)}))
    > rownames(cannot.s2) <- gsub("S\\d\\.","",rownames(cannot.s2))
    > #create comparison table between all samples
    cmb <- combn(ncol(scores2.s)-1,2)
    > #setup data
    > cannot.s2 <- cannot.s2[order(cannot.s2$soleus,cannot.s2$perc),]
    > data.final <- data.final[,rownames(cannot.s2)]
  b. Calculate the median compression ratio (problem 8).
    > #calculate compression ratios between samples using smoothed profiles and a delay strategy
    > scores2.smo.s <- subset(scores2.smo,abs(mean) > 5)
    > bxs <- sapply(rownames(scores2.smo.s), function(dx){
    lns <- lapply(unique(cannot.s2$replicate),function(x){
    ct <- subset(cannot.s2, replicate == x)
    dd <- data.final[dx,rownames(ct)]
    los <- loess(y∼x,data=data.frame(y=dd,x=ct$perc))
    prd <- predict(los,newdata=data.frame(x=seq(0,1,len=5000)))
    return(data.frame(replicate=x,x=seq(0,1,len=5000),y=prd))})
    lns.dat <- sapply(lns,function(x) x$y)
    colnames(lns.dat) <- sapply(lns,function(x) x$replicate [1])
    cors <- sapply(seq(-2500,-1,by=1),function(x){
    res <- c()
    for(i in 1:ncol(cmb)){
    t1 <- lns.dat[x:0,cmb[1,i]]
    t2 <- lns.dat[1:min(c(x+5000,5000)),cmb[2,i]]
    t3 <- lns.dat[x:0,cmb[2,i]]
    t4 <- lns.dat[1:min(c(x+5000,5000)),cmb[1,i]]
    res <- c(res,cor(t1,t2),cor(t3,t4))}
    return(res)})
    cors.up <- cors[seq(1,nrow(cors),by=2),drop=F]
    cors.lo <- cors[seq(2,nrow(cors),by=2),drop=F]
    fix.lo <- apply(cors.lo,1,function(x) seq(0,1,len=5000)[abs(seq(-2500,-1,by=1)[which.max(x)])])
    fix.up <- apply(cors.up,1,function(x) seq(0,1,len=5000)[abs(seq(-2500,-1,by=1)[which.max(x)])])
    fix <- rbind(fix.lo,fix.up)
    colnames(fix) <- apply(cmb,2,paste,collapse="-")
    return(fix)})
    > #across selected proteins to identify compression ratios create a median compression ratio and select the right stretch/compress strategy
    > final.fix <- rowMedians(bxs)
    > final.fix <- matrix(final.fix,nrow=2)
    > colnames(final.fix) <- apply(cmb,2,paste,collapse="-")
    > rownames(final.fix) <- c("lo","up")
    > touse <- final.fix[,grep("1",colnames(final.fix)),drop=F]
    > sol.names <- unique(cannot.s2$replicate)
    > names(sol.names) <- 1:(ncol(scores2.s)-1)
    > cannot.s2$perc2 <- cannot.s2$perc
    for(i in 1:ncol(touse)){
    p <- which.max(touse[,i])
    #p <- 2
    n <- strsplit(colnames(touse)[i],"-")[[1]]
    n <- sol.names[as.character(n)]
    if(p == 1){
    cannot.s2$perc2[which(cannot.s2$replicate == n[2])] <- cannot.s2$perc2[which(cannot.s2$replicate == n[2])]-touse[p,i]
    }else{cannot.s2$perc2[which(cannot.s2$replicate == n[2])] <- cannot.s2$perc2[which(cannot.s2$replicate == n[2])]+touse[p,i]}}
    > ### Evaluate the need for compression ratios between samples END
  c. Create final data (problem 8).
    > ### Create final dataset
    > #helper functions
    > myloess <- function (x, y = NULL, nsigma = 1, newdata=data.frame(x=dis), ...)
    {xy <- xy.coords(x, y)
    x <- xy$x
    x0 <- sort(x)
    y <- xy$y
    nsigma <- as.numeric(nsigma)
    mod <- loess(y ∼ x, ...)
    yfit <- predict(mod, newdata)
    r <- residuals(mod)
    modr <- loess(I(rˆ2) ∼ x, ...)
    sd <- sqrt(pmax(0, predict(modr, newdata)))
    list(model = mod, x = x0, y = yfit, sd = sd, upper = yfit +
    nsigma ∗ sd, lower = yfit - nsigma ∗ sd)}
    > range01 <- function(x){(x-min(x,na.rm=T))/(max(x,na.rm=T)-min(x,na.rm=T))}
    > adjust <- function(x,annot,norange=F){
    x <- x[,rownames(annot)]
    newx <- do.call(cbind,by(t(x),annot$replicate,function(x) t(scale(x))))
    if(!norange)
    newx <- t(apply(newx,1,range01))
    return(newx[,rownames(annot)])}
    > #create final dataset
    > profileDat <- list(
    full.z.2=adjust(data.final[,-match(crem,colnames(data.final))],cannot.use,norange=T),
    full.zr.2=adjust(data.final[,-match(crem,colnames(data.final))],cannot.use))
    > ### Create final dataset END
    ***Note:*** The time required for calculation steps is highly dependent on the dataset because of the following variables: number of proteins within the data set, missing values within the data set, and number of biological replicates (minimum number would be 3 biological replicates). The time required also depends on the CPU. Steps are suitable for parallel processing.

### Bioinformatic analysis: Generation of distance-based networks

**Timing: 120 profiles per day**

This section generates a distance-based network, which performs a delay analysis with the parameter of distance. Individual protein profiles are correlated to each other and shifted to identify proteins with similar profiles.

14. Calculate the distance-delay correlation (problem 9).
  a. Use multiple CPU cores if possible.
    > #use apply with a progressbar
    > library(pbapply)
    > #use igraph libraby for convenience
    > library(igraph)
    > #use multicore where possible
    > library(parallel)
    > #extract the specific dataset to use
    > dd <- profileDat[["full.zr.2"]]
    > #get all pairwise combinations of proteins
    > cmb <- combn(rownames(data),2)
    > cl <- makeCluster(15) #15 cores were used
    > #export the data to the parallel instances
    > clusterExport(cl,c("data","delayc"))
  b. Calculate the delay for all protein profiles.
    > #code that calculates the delay correlation
    > delayc <- function(x,y,delayt=length(x)/10∗4.5,plot=F,method="s",delayx=0.2){
    delayt <- floor(delayt)
    cor1 <- c()
    cor2 <- c()
    for(i in delayt:length(x)){
    tmp <- cor(x[1:i],y[(length(x)-i+1):length(x)],method=method)
    tmp2 <- cor(y[1:i],x[(length(x)-i+1):length(x)],method=method)
    cor1 <- c(cor1, tmp)
    cor2 <- c(cor2, tmp2)
    if(plot){
    par(mfrow=c(1,2))
    plot(x[1:i],ylim=c(min(x,y),max(x,y)),type="l",main=round(tmp,3))
    lines(y[(length(x)-i+1):length(x)])
    plot(y[1:i],ylim=c(min(x,y),max(x,y)),type="l",main=round(tmp2,3))
    lines(x[(length(x)-i+1):length(x)])
    Sys.sleep(delayx)}}
    corr <- c(cor1,rev(cor2))
    #which(corr == max(corr))
    names(corr) <- c(rev(-seq(0,floor(length(corr)/2),length=length(corr)/2)),seq(0,floor(length(corr)/2),length=length(corr)/2))
    corr <- corr[-which(names(corr) == 0)[1]]
    names(corr) <- -floor(length(corr)/2):floor(length(corr)/2)
    return(corr)}
    > #calculate the delays for all proteins
    > dl <- pbapply(cmb,2,function(x) delayc(data[x[1],],data[x[2],],plot=F),cl=cl)
    > #extract those that have the highest correlation
    > dlx2 <- data.frame(t(cmb),delay=apply(dl,2,function(x) as.numeric(rownames(dl)[which.max(x)])),corr=apply(dl,2,max))
  c. Remove proteins with a delay greater than 20.
    > #remove any with a delay greater than 20
    > dlx2.s <- subset(dlx2,delay > -20 & delay < 20)
    > #create the inverse connection, i.e. a -> b and put in b -> a with a switched around delay
    > dlx3 <- rbind(dlx2.s,data.frame(X1=dlx2.s$X2,X2=dlx2.s$X1,delay=dlx2.s$delay∗-1,corr=dlx2.s$corr))
    dlx4 <- subset(dlx3,delay >= 0)
    > rownames(dlx4) <- paste(dlx4[,1],dlx4[,2],sep="-")
    > #use a graph representation for quick access
    > graph <- graph_from_data_frame(dlx4,directed=T)
    > #function to remove unecessary connections
    > #What this does is look at all possible triplet combinations
    > #First it identifies the neighbours in the graph of the 2 nodes passed
    > #Then identifies whether there is an intersect between the two suggesting that other nodes might carry more information than this connection
    > #Then it extracts the data and looks for delays that are greater than 0, and where the sum of delay is below the percentage of dpi added to the current delay. So if the delay is 5 it looks for whether the sum of the delay of the other 2 connections is below that. Also the correlation needs to be on average greater than the current correlation-dpi.
    > #It then returns whether there is a better combination of nodes than the current one (T/F)
    > dpi <- function(x,y,graph=gg,dat=dlx3,dpi=0.1){
    require(igraph)
    x_n <- names(neighbors(graph,x,mode="out"))
    y_n <- names(neighbors(graph,y,mode="in"))
    i_n <- intersect(x_n,y_n)
    cur_edge <- paste(x,y,sep="-")
    alt_edge1 <- paste(x,i_n,sep="-")
    alt_edge2 <- paste(i_n,y,sep="-")
    cur_d <- dat[cur_edge,"delay"]
    if(cur_d < 0)
    return(match(cur_edge,rownames(dat)))
    cur_c <- dat[cur_edge,"corr"]
    alt_d <- cbind(e1=dat[alt_edge1,"delay"],e2=dat[alt_edge2,"delay"])
    alt_c <- cbind(e1=dat[alt_edge1,"corr"],e2=dat[alt_edge2,"corr"])
    alt_d <- cbind(alt_d,s=rowSums(alt_d))
    alt_c <- cbind(alt_c,s=rowMeans(alt_c))
    sel <- which(alt_d[,1] > 0 & alt_d[,2] > 0 & alt_d[,3] < cur_d∗(1+dpi) & abs(alt_c[,3]) >= abs(cur_c)-dpi)
    if(length(sel) > 0){return(T)}else{return(F)}}
    > #another cluster with more cores
    > #cl <- makeCluster(100) #100 core cluster
    > clusterExport(cl,c("graph","dlx4","dpi"))
    > #identify whether the current combination is ideal or whether other combinations exist that are better, TRUE means there is something better, FALSE means there isn't anything better. Hence we would like to extract all FALSE entries
    > res <- pbapply(dlx4,1,function(x) dpi(x[1],x[2],graph=graph,dat=dlx4),cl=cl)
    > #extract FALSE entries
    > dlx4.s <- dlx4[which(!res),]
    > #add an inverse delay for better plotting in cytoscape
    > dlx4.s$delay_inv <- 1/(dlx4.s$delay+1)
    > #write the file as CSV and load into cytoscape
    > write.csv(dlx4.s,"InteractionNetwork_2023.04.21.csv",quote=F)
  d. Perform visualization in cytoscape.

## Expected outcomes

### Cryotome sectioning

**Figure 4:**
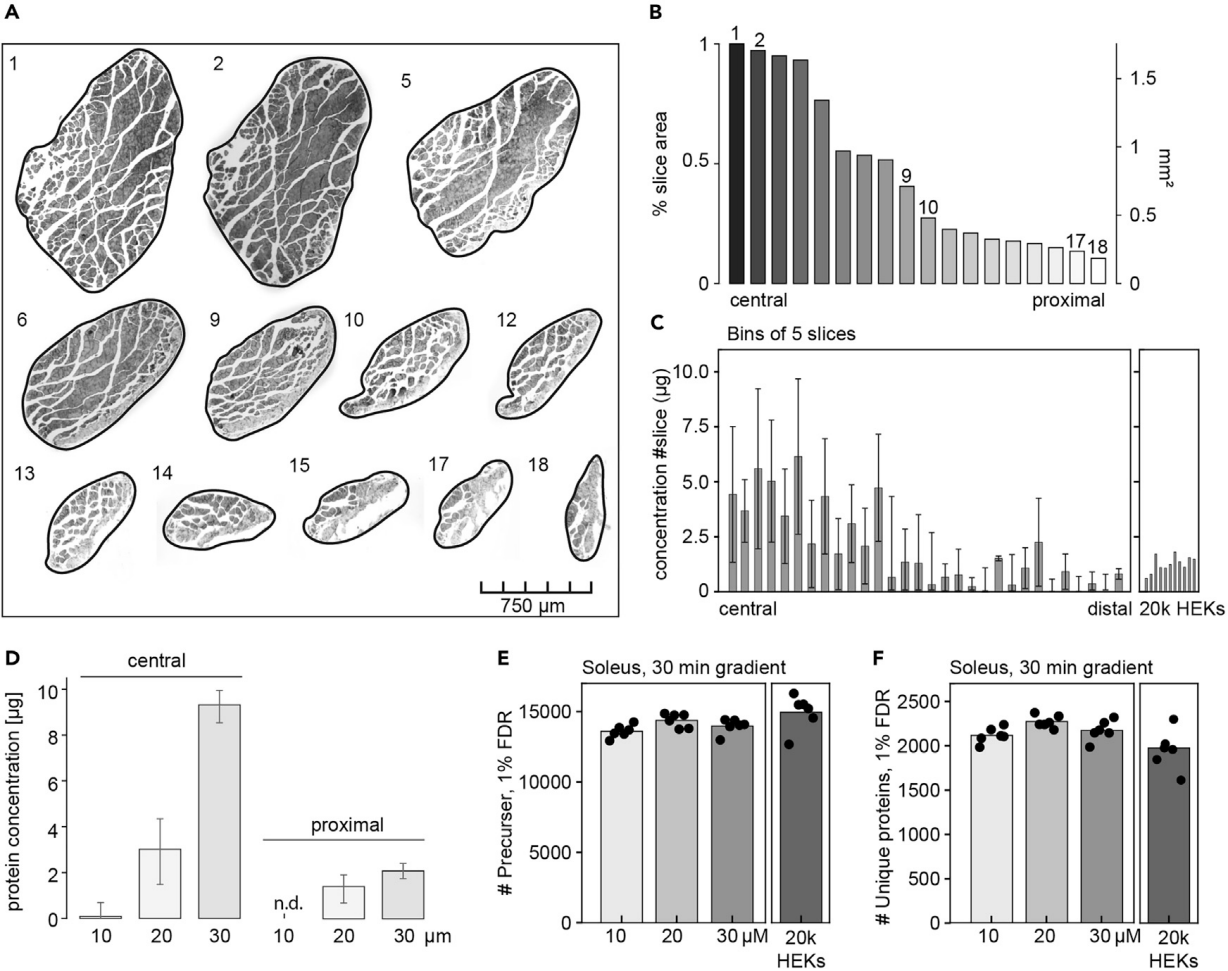
Expected outcomes for muscle sectioning and protein lysis (A) Muscle sections from the central muscle (1) to the proximal end of the muscle. (B) Slice area of sections from central muscle to the proximal end of the muscle (C) and the respective total protein inputvalues ± SEM per section (sections are binned to 5). (D) Total amount of protein input ± SEM per 10‒30 μm-thick section (n = 5). (E) Number of unique peptides and (F) proteins identified per 10‒30 μm-thick section.

Cryo-embedded soleus muscles and consecutive cross-sections prepared from the central to distal areas using a cryotome (Figures 4A and 4B). For tissues with a length of 3.5 mm, there will be 175 consecutive 20 μm-thick sections.

### Tissue lysis and digestion and LC-MS/MS analysis

For a 20 μm-thick tissue section with an area of approximately 1.7 mm^2^, 4 μg of protein can be expected. For tissues with a smaller area, it is recommended to use thicker sections; thinner sections can be used to obtain a higher resolution over the length of the tissue. The distal (tendon) slices have an area of less than ∼0.2 mm^2^ and yield less than 1 μg protein (Figures 4B–4D). Even though the input variances of 10–30 μm are relatively large, the LC-MS/MS performance should be stable and identify in high dynamic ranged tissue around ∼2000 proteins per 20-μm-thick section (Figures 4E and 4F).

### Bioinformatic analysis

Depending on the tissue type, heterogeneity, and sample size, a data set of > 7,000 proteins can be expected. For tissues with high heterogeneity in terms of cell distribution, missing values are to be expected — especially between the different tissue types. Additionally, a variety between tissue lengths can be expected with slightly different protein profile lengths because of biological variability of the skeletal muscle length. Over 3,000 protein profiles can be expected depending on the sensitivity of the instrument.

## Limitations

The protocol may not be ideal or particularly beneficial for all types of tissues. In cases where tissues exhibit low heterogeneity or lack distinct regions, such as in the liver, the advantages of this protocol may be minimal. The effectiveness of the protocol is highly dependent on the resolution required for the specific tissue and its regions. In some instances, limitations due to the sensitivity of the instruments used may exist. Additionally, the depth of proteomic analysis is significantly influenced by the capabilities of the MS instrument employed.

Furthermore, biological variability within a group of replicates (animals) can be more pronounced in these studies compared to other research that focuses on intact tissues. This increased variability can complicate interpretation of the results and potentially obscure meaningful differences. Consequently, careful consideration must be given to the choice of tissue type and the specific requirements of the study to ensure that the application of this protocol is both appropriate and effective.

## Troubleshooting

### Problem 1

The cork mat should be removed very carefully, otherwise the cryo-matrix and the tissue could be damaged.

### Potential solution

• Place enough cryomatrix between the tissue and the cork mat to prevent the tissue from freezing directly to the cork. It is also possible to first fill the lower half of the cryomold with Tissue-Tek and cool the cryomold until the cryomatrix is slightly frozen. Then, position the tissue on top of the frozen cryomatrix, embed the tissue completely with Tissue-Tek, and completely freeze the cyromatrix. Care must be taken here to ensure that the cryomaterial freezes evenly.
• Alternatively, the tissue can also be placed on a polyvinylidene fluoride (PVDF) membrane. By pressing the tissue tightly to the membrane, the tissue is fixed in the correct position.

### Problem 2

Blank MS measurements because of sample loss during transfer or protein extraction.

### Potential solution

• Visually check that all the wells contain a cryosection.
• Perform an additional centrifugation step before adding lysis buffer to move the sections to the bottom of the well.
• As an alternative to Tissue-Tek, formaldehyde-fixed paraffin-embedded tissue (FFPE) can also be used. In this case, the lysis buffer can be added before the section is transferred to the plate. The protein extraction protocol should be adjusted for FFPE sections as described in.^8^

### Problem 3

Magnetic Sera-Mag beads stick to the walls of the 96-well-plate.

### Potential solution

• Lysis buffer can be added several times to the well to wash the beads away from the wall of the well.
• Before the overnight digestion step, shake the 96-well-plates at 1,300 rpm for 10 min at 37°C.

### Problem 4

Protein input variability across the tissue samples. Since label-free quantification (LFQ) works optimally for similar samples, variation in the amount and composition of the skeletal muscle and tendon areas could interfere with the normalization and LFQ quantification.

### Potential solution

• Normalization can be performed by dividing the individual protein intensities by the sum of the protein intensity per slice. This ensures that all protein intensities become relative to the total intensity.
• Normalize to a housekeeping protein. If the tissue heterogeneity is minimal, normalization of the protein intensities to a house-keeping gene like actin or tubulin may be an alternative.
• Spike in heavy peptides. Isotope-labeled peptides, used in the same concentration in all samples and are detected in the MS instruments, can be used to normalize peptide and protein intensities.
• Total ion chromatogram (TIC) normalization: Samples can be measured using very short gradients in MS1 mode. The TIC can be then used to adjust the injection volume.

### Problem 5

Selection of protein profiles with missing values.

### Potential solution

• To generate protein profiles that are as valid and complete as possible, only proteins that were detected in at least 70% of all data points (in this case, slices) should be selected.
• For tissues with high heterogeneity, it is recommended to use a sliding window approach as described in Schmidt et al. The sliding window size can be adapted and can cover 5-20 sections.

### Problem 6

Imputation of missing data requires an adjustment of data points from the adjacent slices for linear modeling.

### Potential solution

• The minimum number of datapoints should not be less than 5 to ensure enough data points are present for imputation.

### Problem 7

We used a bootstrapping approach based on the R package *samr* (^9^) to identify which proteins can be used for alignment between the different muscles. This ensures identification of proteins with strong increasing or decreasing trends that are suitable for alignment. This procedure can be replaced by other approaches.

### Potential solution

• Several statistical approaches have been proposed to identify features that align with various profiles. Alternative examples are: BETR (Bayesian Estimation of Temporal Regulation),^10^ Mutual Information,^11^ correlation,^11^ and ANOVA.^12^

### Problem 8

The protein profiles are not smooth enough.

### Potential solution

• When creating profiles, we use the loess function to generate local average slopes. The span parameter allows for fewer or more samples to be included, which changes the smoothness of the profiles that are generated.
• Alternative smoothing functions such as smooth, Spline, lowess, and approx within R can also be utilized.

### Problem 9

Delays between features are too high.

### Potential solution

• When calculating the delay between two features, the approach shifts one profile against the other. When the number of delays is too high, the number of samples included in the correlation goes towards zero, which often leads to an increase in the correlation towards 1. This issue can be handled with the *delayt* parameter within the *delayc* function.

## Resource availability

### Lead contact

Further information and requests for resources and reagents should be directed to and will be fulfilled by the lead contact, Marcus Krüger (Marcus.krueger@uni-koeln.de).

### Technical contact

Technical questions on executing this protocol should be directed to and will be answered by the technical contact, Luisa Schmidt (luisa.schmidt@uni-koeln.de).

### Materials availability

• This study did not generate new unique reagents.
• Data and code availability statement examples.
• The published article includes all datasets and code generated or analyzed during this study.

### Data and code availability

The accession number for the Dataset 'Dataset1_sorted.xlsx' reported in this paper is deposited at Zenodo: https://doi.org/10.5281/zenodo.10678005.

## Acknowledgments

This work was supported by the Cologne Cluster of Excellence on Cellular Stress Responses in Aging-associated Diseases (CECAD) EXC 299/2, the RELOC graduate school GRK 2550 (DFG 411422114), and FOR2722/2 (Krüger_384170921/TP4, KR 3788/8-2). In addition, this work was supported by a major investment grant from the Deutsche Forschungsgemeinschaft (INST 216/1163-1 FUGG and INST 216/1020-1 FUGG) and the JPND2019-466-146 grant.

## Author contributions

Conceptualization, L.S. and M.K.; methodology, L.S. and M.K.; software, L.S. and P.A.; formal analysis, L.S. and P.A.; investigation, L.S.; resources, M.K.; visualization, L.S.; supervision and project administration, M.K.; funding acquisition, M.K.

## Declaration of interests

The authors declare no competing interests.
